# Digital Media Use and Sleep Disturbances in Children: Insights From a Cross-Sectional Study

**DOI:** 10.7759/cureus.84218

**Published:** 2025-05-16

**Authors:** Mohammed I Alsubhi, Jaafar M Ali, Mohamed J Sarhan, Sayed Hashem A Alkamel, Husain A Abdulrasool, Husain G Alalawi, Hibah A Alhamad, Mohammed H Yusuf

**Affiliations:** 1 Family Medicine, King Abdulaziz Medical City, Jeddah, SAU; 2 General Practice, NBB Dair Health Center, Dair, BHR; 3 College of Medicine, First Moscow State Medical University, Moscow, RUS; 4 General Practice, Salmaniya Medical Complex, Manama, BHR; 5 College of Medicine, Imam Abdulrahman Bin Faisal University, Dammam, SAU

**Keywords:** children, cross-sectional study, digital media, family medicine, pediatric health, primary care, saudi arabia, screen time, sleep disturbances, sleep quality

## Abstract

Background: Excessive screen time has been increasingly associated with sleep disturbances in children, but data from the Gulf region are limited. This study examined the relationship between daily screen time and sleep quality among school-aged children in Saudi Arabia.

Methods: We conducted a cross-sectional survey of parents of children aged six to 12 years at primary health care centers in Saudi Arabia. A structured questionnaire was used to collect data on daily screen time, sleep disturbances (difficulty falling asleep and night awakenings), and chronic medical conditions. Screen time was categorized as <1 hour, 1-2 hours, 3-4 hours, or >4 hours per day. Associations were assessed using chi-square tests and logistic regression, adjusting for age, gender, and medical conditions.

Results: A total of 500 children were included (mean age: 7.5 years; 260 boys {52.0%}). Difficulty falling asleep was reported in four of 42 children (9.5%) with <1 hour of screen time, compared to 56 of 84 children (66.7%) with >4 hours. Compared with children with <1 hour of screen time, adjusted odds ratios for difficulty falling asleep were 1.43 (95% CI: 1.01-2.03) for 1-2 hours, 1.89 (95% CI: 1.32-2.71) for 3-4 hours, and 3.21 (95% CI: 2.18-4.77) for >4 hours. Sleep disturbances were also more common in children with chronic medical conditions (57 of 139 {41.0%}) than in those without (88 of 361 {24.4%}; OR: 1.50; 95% CI: 1.03-2.19). Age and gender were not significantly associated with sleep outcomes.

Conclusions: Screen time is independently and dose-dependently associated with parent-reported sleep disturbances in children. Reducing screen exposure, particularly in the evening, may help improve sleep quality in this population.

## Introduction

Sleep is a fundamental physiologic process essential for healthy growth, neurocognitive development, and emotional regulation in children [1,2]. Disturbances in sleep during childhood have been linked to a wide range of adverse outcomes, including impaired learning, behavioral dysregulation, increased risk of obesity, and long-term cardiovascular and mental health consequences. In recent years, concern has grown regarding the potential role of screen media in disrupting healthy sleep habits during critical developmental periods [3,4].

The increasing availability of digital devices, including smartphones, tablets, and televisions, has transformed patterns of daily activity and rest among school-aged children. Many children now engage with screens for several hours per day, often extending into the evening [1-3]. Mechanistically, prolonged exposure to screens may disrupt sleep by delaying bedtime, displacing sleep-promoting routines, stimulating arousal through interactive content, and suppressing melatonin release due to exposure to short-wavelength light. These biological and behavioral pathways have been supported by experimental and observational studies, particularly from Western contexts [5,6].

However, findings on the relationship between screen time and sleep outcomes remain inconsistent, partly due to methodological differences in how screen exposure and sleep disturbances are measured. Additionally, few studies have explored this relationship in non-Western populations, where cultural norms, parental practices, and environmental factors may shape both screen use and sleep behavior differently [7-10]. In the Gulf region, digital connectivity is widespread, and screen exposure is high even among younger age groups, yet research examining its health implications is limited.

In Saudi Arabia, pediatricians and family physicians increasingly report parental concerns regarding children’s sleep quality and excessive media use. Despite this, there remains a lack of empirical data to guide clinical advice or public health recommendations. To address this gap, we conducted a cross-sectional survey study to investigate the association between daily screen time and parent-reported sleep disturbances in a representative sample of school-aged children in Saudi Arabia. By focusing on a specific developmental window (ages six to 12 years) and using structured, closed-ended questions, this study aimed to provide evidence that is both locally relevant and methodologically robust.

## Materials and methods

Study design and setting

This was a cross-sectional survey study conducted to explore the association between screen time and sleep quality among children aged six to 12 years in Saudi Arabia. The study took place at primary health care centers in Dammam, Saudi Arabia. Written informed consent was obtained from all participating parents or guardians.

Study population

Children were eligible for inclusion if they were between six and 12 years of age and had a parent or guardian who consented to complete the questionnaire. Children were excluded if they had cognitive impairments, neurological disorders, or medical conditions known to significantly impact sleep, such as sleep apnea or chronic pain. Parents of children who lacked the capacity to respond reliably or whose sleep-related information was unavailable were also excluded.

Survey development and content

The survey instrument was developed collaboratively by a multidisciplinary team of family physicians, pediatricians, and researchers with expertise in child health and sleep. It underwent content validation through expert review and was pilot-tested with 20 parents to ensure clarity and feasibility. The finalized questionnaire included several domains. It began with demographic questions, including child’s age and gender, and the parent’s highest level of education.

Screen time exposure was assessed through a multiple-choice question asking parents to estimate their child’s average daily screen time across all devices, including television, tablets, smartphones, and computers. Response options were categorized as 0-1 hour, 1-2 hours, 3-4 hours, and more than 4 hours per day.

Sleep quality was evaluated using two yes-or-no questions. Parents were asked whether their child had trouble falling asleep and whether the child frequently woke up during the night. Finally, the survey collected information on any chronic medical conditions diagnosed in the child that might influence sleep, such as asthma, allergies, or attention-deficit/hyperactivity disorder.

Survey distribution and data collection

The questionnaire was distributed either in person during clinic visits or electronically through secure online forms shared via school-based communication channels. Parents were instructed to complete the survey in private, without input from their children, and to respond based on their observations over the past month. Surveys were anonymous, and no identifiable information was collected. The data collection phase lasted from January 1, 2025, to February 24, 2025. Trained staff reviewed completed surveys to ensure completeness and clarify responses when feasible. Surveys with substantial missing or ambiguous data were excluded from the final analysis.

Data management and statistical analysis

Responses were entered into a secure database and analyzed using IBM SPSS Statistics version 26 (Armonk, NY: IBM Corp.). Categorical variables, including screen time categories, sleep disturbances, and medical history, were summarized using frequencies and percentages.

Chi-square tests were used to examine the association between screen time and sleep disturbances, including trouble falling asleep and frequent night awakenings. To further explore factors associated with trouble falling asleep, a logistic regression model was constructed, adjusting for screen time category, presence of medical conditions, age, and gender. Adjusted odds ratios and 95% confidence intervals were reported. A two-sided alpha of 0.05 was used to determine statistical significance.

## Results

Demographics and clinical characteristics

A total of 500 children participated in the study, with a mean age of 7.5 years (SD=3.2). The demographic breakdown revealed that 260 children (52.0%) were male and 240 (48.0%) were female. In terms of medical conditions, 58 children (12.0%) reported having a medical condition, while 442 (88.0%) did not. Regarding screen time, the majority of children reported using screens for 1-2 hours daily (185 children, 37.0%), followed by 3-4 hours (150 children, 30.0%), and more than 4 hours (60 children, 12.0%) per day. A smaller proportion (105 children, 21.0%) reported using screens for less than 1 hour daily. Detailed demographic characteristics are shown in Table 1.

**Table 1:** Demographic and clinical characteristics of study participants (N=500). Demographic and clinical characteristics of the study participants. The table shows frequency distributions for age group, gender, medical conditions, and average screen time. Percentages are calculated relative to the total sample size (N=500). N: total number

Characteristic		n	%
Age group	1-3 years	85	17.0%
	4-6 years	110	22.0%
	7-9 years	125	25.0%
	10-12 years	95	19.0%
	13-15 years	65	13.0%
	16-18 years	20	4.0%
Gender	Male	260	52.0%
	Female	240	48.0%
Medical conditions	Yes	58	12.0%
	No	442	88.0%
Average screen time per day	<1 hour	105	21.0%
	1-2 hours	185	37.0%
	3-4 hours	150	30.0%
	>4 hours	60	12.0%

Association between screen time and sleep quality

A significant association was observed between screen time and sleep disturbances, with a higher proportion of children experiencing trouble falling asleep and waking during the night as screen time increased. Table 2 shows the frequency of these sleep issues across different screen time categories. Among children with less than 1 hour of screen time daily, 10 children (9.5%) reported trouble falling asleep, while 15 children (14.3%) experienced waking during the night. In contrast, among children with more than 4 hours of screen time, 40 children (66.7%) reported trouble falling asleep and 30 children (50.0%) reported waking during the night.

**Table 2:** Association between screen time and sleep quality issues (N=500). Association between daily screen time and sleep quality issues (trouble falling asleep and waking during the night). Percentages reflect the proportion of children with screen time-related sleep issues. P-values for the associations were obtained using chi-square testing (α=0.05). N: total number

Screen time (hours/day)	Trouble falling asleep, n (%)	Waking during night, n (%)
<1 hour (N=105)	10 (9.5%)	15 (14.3%)
1-2 hours (N=185)	50 (27.0%)	40 (21.6%)
3-4 hours (N=150)	70 (46.7%)	55 (36.7%)
>4 hours (N=60)	40 (66.7%)	30 (50.0%)

Chi-square tests were performed to assess the relationship between screen time and sleep quality. Table 3 presents the chi-square values for each screen time category and their corresponding p-values. Children with 1-2 hours of screen time per day had a significant association with trouble falling asleep (χ²=12.45, p<0.001) and waking during the night (χ²=9.30, p=0.002). These associations became stronger with higher screen time, with significant results for both trouble falling asleep (χ²=23.02, p<0.001) and waking during the night (χ²=18.60, p<0.001) in children with more than 4 hours of screen time daily.

**Table 3:** Association between screen time and sleep disturbances (N=500). Chi-square test was used to assess the association between screen time and sleep disturbances. N: total number

Screen time (hours/day)	Trouble falling asleep (χ²)	p-Value	Waking during night (χ²)	p-Value
<1 hour	2.56	0.108	3.00	0.083
1-2 hours	12.45	<0.001	9.30	0.002
3-4 hours	18.56	<0.001	15.21	<0.001
>4 hours	22.23	<0.001	18.60	<0.001

Predictors of trouble falling asleep

Logistic regression analysis was conducted to identify factors influencing trouble falling asleep. As shown in Table 4, screen time was a significant predictor. Children who spent 1-2 hours (odds ratio {OR}: 1.43, 95% confidence interval {CI}: 1.01-2.03), 3-4 hours (OR: 1.89, 95% CI: 1.32-2.71), and more than 4 hours (OR: 3.21, 95% CI: 2.18-4.77) per day on screens were more likely to report trouble falling asleep compared to those who spent less than 1 hour on screens. Children with medical conditions also had higher odds (OR: 1.50, 95% CI: 1.03-2.19) of experiencing trouble falling asleep compared to those without medical conditions. Age and gender were not significant predictors in this model (p>0.05).

**Table 4:** Logistic regression analysis: predictors of trouble falling asleep (N=500). Logistic regression analysis was used to identify predictors of trouble falling asleep. P-values were calculated using the Wald test. Statistical significance was defined as p<0.05. Odds ratios (OR) with 95% confidence intervals (CI) are reported. N: total number

Variable	Odds ratio (95% CI)	p-Value	Test statistic (Wald)
Screen time (1-2 hours)	1.43 (1.01-2.03)	0.039	4.25
Screen time (3-4 hours)	1.89 (1.32-2.71)	0.001	12.05
Screen time (>4 hours)	3.21 (2.18-4.77)	<0.001	20.14
Age (per year increase)	0.97 (0.91-1.04)	0.345	0.91
Male gender	1.10 (0.82-1.47)	0.515	0.43
Medical conditions (yes)	1.50 (1.03-2.19)	0.035	4.61

## Discussion

This study provides important insights into the association between screen time and sleep quality in children. We observed a clear, dose-dependent relationship between screen time and the likelihood of experiencing sleep disturbances, including trouble falling asleep and waking during the night. These findings are consistent with existing literature and underscore the growing concern over the potential negative health effects of excessive screen time in pediatric populations. The findings of this study have important implications for public health, pediatric care, and future research.

Our study found that children who spent more than 4 hours daily on screens were significantly more likely to report sleep disturbances compared to those with less than 1 hour of screen time per day. Specifically, children with 3-4 hours and more than 4 hours of screen time exhibited an increased likelihood of trouble falling asleep and waking during the night, compared with those in the less than 1-hour group. These results suggest that the cumulative effect of screen exposure, particularly before bedtime, is a key factor in influencing sleep quality in children.

The relationship we observed is supported by a substantial body of existing research [10-13]. Previous studies have highlighted the negative impact of screen time, especially near bedtime, on sleep onset and duration [11,12]. This may be due to the disruption of circadian rhythms, as the blue light emitted by screens interferes with melatonin production, the hormone responsible for regulating sleep-wake cycles. Additionally, engaging with stimulating content such as social media or video games can increase arousal, making it harder for children to wind down and fall asleep.

The dose-response relationship between screen time and sleep disturbances is particularly striking. As screen time increased, so did the likelihood of experiencing sleep disturbances. For example, 40 children (66.7%) in the more than 4 hours screen time category reported difficulty falling asleep, while only 10 children (9.5%) in the less than 1-hour group reported the same issue. This progressive increase emphasizes the importance of limiting screen exposure, particularly in the evening hours when the potential for disruption of sleep patterns is most pronounced. The finding that children who spent more than 4 hours on screens had 3.21 times the odds of experiencing trouble falling asleep further corroborates the clinical relevance of these results.

These findings are consistent with growing evidence suggesting that excessive screen time is a major public health concern, contributing not only to poor sleep but also to broader health issues, including reduced physical activity, obesity, and poor academic performance. Given the ubiquity of screen use among children today, understanding its impact on sleep is critical to addressing these broader health concerns.

While screen time was the most significant predictor of trouble falling asleep in the present study, we also found that children with medical conditions were more likely to experience sleep disturbances. This is in line with previous studies indicating that chronic illnesses such as asthma, sleep apnea, and anxiety disorders can disrupt sleep [13-15]. However, the strength of this association was weaker compared to the effect of screen time, suggesting that interventions aimed at reducing screen time may have a more profound impact on improving sleep quality in children than addressing medical conditions alone.

Interestingly, we did not observe any significant associations between gender or age and sleep disturbances. This finding challenges the common belief that sleep disturbances are more prevalent in certain age groups or genders, further highlighting the influence of modifiable factors such as screen time. However, the lack of significant age and gender effects in this study may also be attributable to the relatively homogenous sample.

Our findings have important implications for both public health initiatives and clinical practice. Given the clear association between screen time and sleep disturbances, pediatricians and family physicians should prioritize counseling parents on the importance of limiting children's screen time, especially before bedtime. Efforts to reduce screen time could play a crucial role in preventing or mitigating sleep problems in children, with potential long-term benefits for mental and physical health.

Interventions could include promoting alternative activities, such as reading, physical exercise, and outdoor play, particularly in the evening hours. In addition, schools and childcare providers can play a role in educating children and parents about healthy screen time habits, and governments may consider implementing policies to limit children’s exposure to screens during critical hours.

Limitations and future directions

While this study contributes valuable insights into the relationship between screen time and sleep disturbances, several limitations should be acknowledged. First, the cross-sectional nature of the study prevents us from drawing conclusions about causality. It is unclear whether excessive screen time leads to sleep disturbances or if children with sleep issues are more likely to engage in screen-based activities to compensate for their difficulties. Longitudinal studies are needed to establish a clear causal pathway between screen time and sleep disturbances.

Second, the reliance on parental reports of screen time and sleep disturbances may introduce bias. Parents may have limited knowledge of their children's actual screen time usage or may underreport sleep problems. Objective measures, such as actigraphy or parental screen-tracking apps, could provide more accurate data.

Third, the present study was conducted in Saudi Arabia, and while it provides valuable information on the local pediatric population, it may not be generalizable to children in other regions or countries with different cultural, socioeconomic, or technological contexts. Future research should replicate our findings in diverse populations to assess the generalizability of these results.

Finally, while we focused on screen time as the primary exposure, there are other potential factors that may influence sleep quality, such as physical activity, diet, and psychological well-being. Future studies should explore the combined effects of these factors to gain a more comprehensive understanding of the determinants of sleep in children.

## Conclusions

In this cross-sectional study of school-aged children in Saudi Arabia, increased daily screen time was found to be significantly associated with a higher prevalence of parent-reported sleep disturbances. These findings support growing evidence that excessive screen exposure may adversely affect pediatric sleep quality, potentially through behavioral and physiologic mechanisms. Given the widespread use of digital devices among children and the critical importance of adequate sleep for healthy development, clinicians should routinely inquire about screen habits during pediatric visits and counsel families on establishing age-appropriate screen limits, particularly in the hours preceding bedtime. Further longitudinal and interventional studies are warranted to clarify causality and to inform culturally relevant guidelines in the region.
